# Consumption of *Bifidobacterium lactis* Bi-07 by healthy elderly adults enhances phagocytic activity of monocytes and granulocytes

**DOI:** 10.1017/jns.2013.31

**Published:** 2014-01-02

**Authors:** Sujira Maneerat, Markus J. Lehtinen, Caroline E. Childs, Sofia D. Forssten, Esa Alhoniemi, Milin Tiphaine, Parveen Yaqoob, Arthur C. Ouwehand, Robert A. Rastall

**Affiliations:** 1 Department of Food and Nutritional Sciences, University of Reading, Reading RG6 6AP, UK; 2 Active Nutrition, DuPont Nutrition and Health, Danisco Sweeteners OY Sokeritehtaantie 20, 02460 Kantvik, Finland; 3 Pharmatest Services Ltd, Itäinen Pitkäkatu 4 C, FI-20520Finland

**Keywords:** Clinical trials, Elderly adults, Probiotics, Prebiotics, Bi-07, *Bifidobacterium animalis* subsp. *lactis* Bi-07, GOS, galacto-oligosaccharide, IFN, interferon, LPS, lipopolysaccharide, ROS, reactive oxygen species

## Abstract

Elderly adults have alterations in their gut microbiota and immune functions that are associated with higher susceptibility to infections and metabolic disorders. Probiotics and prebiotics, and their synbiotic combinations are food supplements that have been shown to improve both gut and immune function. The objective of this randomised, double-blind, placebo-controlled, cross-over human clinical trial was to study immune function and the gut microbiota in healthy elderly adults. Volunteers (*n* 37) consumed prebiotic galacto-oligosaccharides (GOS; 8 g/d), probiotic *Bifidobacterium lactis* Bi-07 (Bi-07; 10^9^ colony-forming units/d), their combination (Bi-07 + GOS) and maltodextrin control (8 g/d) in four 3-week periods separated by 4-week wash-out periods. Immune function was analysed by determining the phagocytic and oxidative burst activity of monocytes and granulocytes, whole-blood response to lipopolysaccharide, plasma chemokine concentrations and salivary IgA levels. Gut microbiota composition and faecal SCFA content were determined using 16S ribosomal RNA fluorescence *in situ* hybridisation and HPLC, respectively. Primary statistical analyses indicated the presence of carry-over effects and thus measurements from only the first supplementation period were considered valid. Subsequent statistical analysis showed that consumption of Bi-07 improved the phagocytic activity of monocytes (*P* < 0·001) and granulocytes (*P* = 0·02). Other parameters were unchanged. We have for the first time shown that the probiotic Bi-07 may provide health benefits to elderly individuals by improving the phagocytic activity of monocytes and granulocytes. The present results also suggest that in the elderly, the effects of some probiotics and prebiotics may last longer than in adults.

Of the population of Europe, 20 % are elderly (aged > 65 years) and this is predicted to increase to 25 % by 2020 according to the WHO. As individuals age, changes to the physiology and function of the gastrointestinal tract and immune system status occur^(^^1^^)^. These changes are associated with increased susceptibility to infections, metabolic disorders and frailty that have significant impact on the quality of life in elderly individuals and healthcare costs to society.

Although age-related changes have been shown in the composition, biodiversity and metabolic activities of the gut microbiota, clear patterns of changes are still obscure due to the impact of the environment and host on the microbiota^(^^2^^–^^4^^)^. For example, the amount of *Bacteroides* in the intestine has been shown to both increase and decrease in elderly subjects depending on the population studied^(^^5^^–^^7^^)^. It is, however, well established that with age the amount of facultative anaerobes increases, such as opportunistic pathogens found in Proteobacteria and Bacilli^(^^5^^–^^9^^)^. Also, the number and diversity of beneficial bifidobacteria have been shown to decline in some studies, indicating that a detrimental shift in the balance of microbial species occurs with ageing^(^^1^^,^^4^^)^.

Changes in the microbiota of the elderly are associated with changes in the immune system status characterised by higher production of pro-inflammatory cytokines^(^^3^^)^. It was recently shown that higher amounts of Bacilli and Proteobacteria in the intestine are associated with increased IL-6 and IL-8 plasma levels in the elderly^(^^3^^)^. Despite the increased levels of pro-inflammatory cytokines, it seems that the reactivity of the innate and adaptive immune systems in the elderly is poorer. *In vivo* these findings are perhaps best highlighted by low vaccination responses that lead to higher susceptibility to infections^(^^10^^,^^11^^)^. On a mechanism level, it has been shown that ageing decreases toll-like receptor (TLR) signalling. For example, lipopolysaccharide (LPS) signalling through TLR4 is impaired, leading to decreased cytokine production and immune function^(^^12^^)^ that could explain the reduced phagocytic capacity of neutrophils in the elderly^(^^11^^,^^13^^)^.

An appealing approach to modulate gut microbiota, poor immune response and detrimental effects of the ageing population is through the use of dietary interventions that have an impact on both the gut microbiota and immune function. Probiotics and prebiotics are widely accepted nutritional supplements that have beneficial effects on both microbiota composition and potentially the immune system in the elderly^(^^14^^–^^16^^)^. Probiotics were defined in 2001 by an FAO/WHO workgroup as ‘live microorganisms which when administered in adequate amounts confer a health benefit on the host’. A prebiotic is defined as ‘a selectively fermented ingredient that results in specific changes in the composition and/or activity of the gastrointestinal microbiota, thus conferring benefit(s) upon host health’^(^^17^^)^. Prebiotics are complex oligosaccharides such as galacto-oligosaccharides (GOS), inulin and fructo-oligosaccharides that are preferentially fermented by health-positive bacteria^(^^18^^–^^21^^)^. This leads to changes in the metabolism of the microbiota and in higher intestinal concentrations of beneficial SCFA^(^^15^^)^. Only a few clinical trials have compared the effects of probiotics, prebiotics and their synbiotic combinations in a single trial. In a recent study it was concluded that changes to microbiota were different in a resistant starch and *Bifidobacterium lactis* synbiotic group than in prebiotic or probiotic groups in patients with colorectal cancer^(^^22^^)^. Another trial concluded that synbiotic (psyllium and *B. longum*) administration improved the quality of life of ulcerative colitis patients and decreased plasma C-reactive protein, but this was not observed in probiotic or prebiotic groups^(^^23^^)^. In addition, in probiotic and prebiotic groups there were independent effects on emotional and bowel function. These studies suggest that synbiotic effects may not be additive, although a study where GOS, *B. lactis* Bb-12 and their synbiotic were administered to healthy adults suggested that the synbiotic increases intestinal bifidobacteria numbers compared with single products^(^^24^^)^.

Consumption of probiotic *B. lactis* strains and GOS has been shown to increase the number of bifidobacteria and to improve phagocytic activity in elderly adults (*B. lactis* HN019 and *trans*-GOS)^(^^24^^–^^26^^)^, indicating that consumption of *B. lactis*, GOS and their synbiotic combination could improve health. This clinical trial aims to study the impact of probiotic *B. animalis* subsp. *lactis* Bi-07 (Bi-07), prebiotic GOS, synbiotic Bi-07 + GOS and maltodextrin control on gut microbiota and the immune system in healthy elderly adults. The study was designed to follow supplementation-induced changes in microbiota by analysing marker bacterial groups and SCFA from faecal samples. Changes in inflammatory status and immune system responsiveness by the supplementation were analysed by measuring the concentration of inflammatory chemokines in plasma and the responsiveness of isolated peripheral blood mononuclear cells to LPS, respectively. Furthermore, phagocytic and oxidative burst activity of monocytes and granulocytes was analysed to associate changes in microbiota and immune cell responsiveness with the function of phagocytic cells.

## Experimental methods

### Trial design

**Fig. 1.**
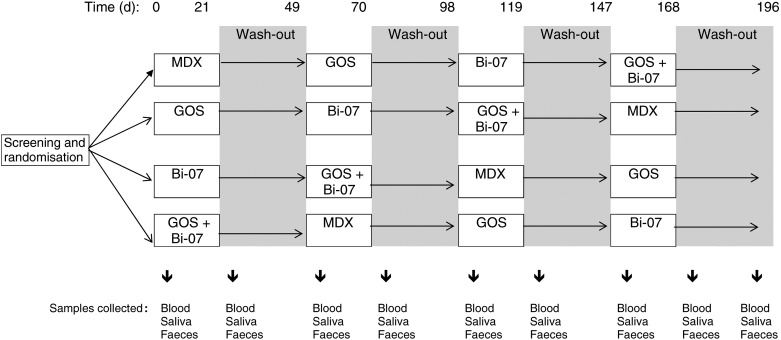
Schematic overview of the study design. MDX, maltodextrin; GOS, galacto-oligosaccharides; Bi-07, *Bifidobacterium animalis* subsp. *lactis* Bi-07.

A total of forty-one healthy elderly volunteers were screened for inclusion to trial. Of these, forty were found to be eligible for the study and were randomly divided into four groups. A double-blinded, placebo-controlled, randomised cross-over study was designed where volunteers received maltodextrin (8 g/d; Syral), prebiotic GOS (8 g/d; Danisco), probiotic (Bi-07; 10^9^ colony-forming units/d; Danisco) and synbiotic GOS + Bi-07 (8 g GOS/d and 10^9^ colony-forming units Bi-07/d). The daily portions were based on dose–response clinical studies showing that an 8 g portion of GOS has a bifidogenic effect^(^^27^^)^ and that 10^9^ colony-forming units of *B. lactis* induce changes in elderly microflora^(^^28^^)^. Volunteers were randomised to groups that consumed the study products in a predefined order: group 1, prebiotic–synbiotic–maltodextrin–probiotic; group 2, synbiotic–maltodextrin–probiotic–prebiotic; group 3, maltodextrin–probiotic–prebiotic–synbiotic; group 4, probiotic–prebiotic–synbiotic–maltodextrin. Supplements were provided for 21 d, with a 28 d wash-out period that has been effective in previous studies with *B. lactis* and GOS^(^^24^^,^^25^^,^^27^^)^ (Fig. 1). Compliance was not measured. The trial was registered at Clinicaltrials.gov as NCT01586247.

### Volunteers

The present study was conducted according to guidelines laid down in the Declaration of Helsinki and all procedures involving human subjects were approved by the Research Ethics Committee of the University of Reading. Written informed consent was obtained from all subjects. Volunteers were recruited from the Reading area. Inclusion criteria were: a signed consent form, age over 60 years, not in residential care, and good general health as determined by medical questionnaires. Exclusion criteria included evidence of physical or mental disease or planned major surgery or use of antibiotics within the previous 6 months. Subject characteristics are described in Table 1. Volunteers attended study appointments before and after each treatment and wash-out period. At study appointments, anthropometric measurements were recorded (weight, blood pressure, waist circumference) and volunteers provided a fasted blood sample (collected into heparinised tubes), and samples of saliva and faeces (Fig. 1).

**Table 1.** Baseline characteristics of volunteers in a double-blind, placebo-controlled, randomised cross-over study of a candidate prebiotic (galacto-oligosaccharides; GOS; 8 g/d), probiotic (*Bifidobacterium animalis* subsp. *lactis* Bi-07 (Bi-07); 10^9^ colony-forming units/d) or synbiotic (GOS + Bi-07) (Mean values with their standard errors, or number of subjects)

	Maltodextrin (*n* 9)		Prebiotic (*n* 9)		Probiotic (*n* 8)		Synbiotic (*n* 10)		
	Mean	sem	Mean	sem	Mean	sem	Mean	sem	*P*
Sex (*n*)									
Male	4		3		5		5		
Female	5		6		3		5		0·69
Age (years)	63·0^a^	0·5	70·7^b^	0·69	64·6^a^	0·5	70·5^b^	1·3	<0·001
Weight (kg)	76·5	4·5	74·5	3·3	73·8	5·4	75·0	4·3	0·98
BMI (kg/m^2^)	25·6	1·3	28·4	1·1	25·6	1·2	25·2	1·4	0·27
Plasma cholesterol (mm)	5·8	0·5	5·6	0·3	5·3	0·2	6·0	0·4	0·66

^a,b^ Mean values within a row with unlike superscript letters were significantly different (*P* < 0·05).

### Study products

The GOS product was synthesised at 55°C (pH 5·0) by 50 acid lactase units (ALU) β-galactosidase-1/g from *Aspergillus oryzae* GC288 using 55 % (w/v) lactose (Dairy Crest) as a substrate. The GOS product yield was 32·45 % (w/w). The product had predominantly Gal(β1–6), Gal(β1–4) and Gal(β1–3) linkages. Bi-07 (ATCC SD5220; American Type Culture Collection) was produced according to good manufacturing practice at the Madison plant by Danisco. The maltodextrin was generously provided by Syral. The products were consumed daily by the subjects as provided in individual daily dose sachets in powder form.

### Sample size determination and randomisation

The study group size was estimated by least standardised difference (LSD) using expected changes in the microbial population and faeces as the primary outcome. On the basis of a 5 % significance level, a power of 95 % for detecting the main effect and two-factor interactions, with a standardised size effect of 0·85 or more, a sample size of eight per treatment per group was required. A total of ten per group were included in the study to allow for potential study ‘drop-out’. Blinding was done by Danisco and randomisation to study groups 1–4 by the investigators.

### Statistical analysis

The data from the cross-over study were analysed using linear fixed-effects models with fixed effect terms for the presence/absence of prebiotic/probiotic treatment and their interaction (i.e. a 2 × 2 factorial approach), and a baseline regression coefficient accounting for individual baseline differences between the subjects. Based on the residual analysis, some variables were transformed using logarithmic, square root or power transformations. All the treatments related to statistically significant factors in the linear models (*P* < 0·05) were further statistically compared using contrasts for the linear models. The number of contrasts depended on the statistically significant terms, and the obtained *P* values were adjusted for multiple comparisons using a single-step algorithm.

The linear model analyses were conducted with R: A Language and Environment for Statistical Computing (version 2.14.2; R Development Core Team) using packages nlme: Linear and Nonlinear Mixed Effects Models (version 3.1-103; J. Pinheiro, D. Bates, S. DebRoy, D. Sarkar and R Development Core Team), and multcomp (version 1.2.12)^(^^29^^)^.

### Phagocytosis and oxidative burst

Phagocytosis and oxidative burst by monocytes and granulocytes were determined in fresh (4–6 h) heparinised whole-blood samples using PHAGOTEST^®^ and BURSTTEST^®^ (ORPEGEN Pharma) in accordance with the manufacturer's instructions. *Escherichia coli*, provided by the manufacturer, were opsonised with complement and immunoglobulins using pooled sera, and were labelled with fluorescein isothiocyanate (FITC). In brief, *E. coli* were incubated for 10 min in heparinised whole blood at 37°C. Phagocytosis was stopped by placing the sample on ice and by adding a solution that quenches extracellularly bound FITC–*E. coli*. Erythrocytes were lysed and sample washed. Using a flow cytometer (FACSCalibur, BD Biosciences), monocytes and granulocytes were identified based upon their characteristic appearance on a FSC/SSC (forward scatter *versus* side scatter) plot. The percentages of phagocytes that had ingested bacteria (fluorescent/total) and had phagocytic activity (number of bacteria per cell as determined by mean fluorescence intensity) were determined.

In BURSTTEST^®^, complement and immunoglobulin-opsonised *E. coli* as provided by the manufacturer were incubated with heparinised whole blood for 10 min at 37°C. Fluorogenic dihydrorhodoamine-123, which reacts with reactive oxygen species (ROS), was added to the sample and incubated for 10 min at 37°C. The reaction was stopped by adding an erythrocyte-lysing solution. After washing, the monocytes and granulocytes were identified by means of an FSC/SSC plot using a flow cytometer (FACSCalibur; BD Biosciences). The production of reactive oxygen metabolites within monocytes and granulocytes was determined by counting the percentage of fluorescent cells and their mean fluorescence intensity, which correlates with ROS production.

### Immune assays

Cytokine and chemokine concentrations were determined using commercially available bead-arrays from BenderMedSystems which both utilise sandwich ELISA technology. All immune assays were conducted to conform to an intra-assay CV of <10 % and an inter-assay CV of <20 %. Sample concentrations were calculated using an eight-point standard curve.

LPS-stimulated cytokine production was analysed in 1/10 diluted whole blood. Roswell Park Memorial Institute (RPMI)-1640 media containing HEPES and l-glutamine (Lonza) was used, to which streptomycin (1 mg/ml) and penicillin (62·5 µg/ml) were added. Cultures were incubated for 24 h in the presence of LPS from *E. coli* O111:B4 (1 µg/ml; Sigma Aldrich). Culture supernatant fractions were assessed for cytokine production using a Th1/Th2 cytokine array (Bender MedSystems) in accordance with the manufacturer's instructions. This array includes interferon (IFN)-γ, IL-1β, IL-2, IL-4, IL-5, IL-6, IL-8, IL-10, IL-12p70, TNF-α and TNF-β. The levels of IL-2, IL-4, IL-5, IL-12p70, and TNF-β were below detection levels in most of the samples (data not presented).

Plasma chemokines were assessed using a human chemokine 6-plex kit (Bender MedSystems) in accordance with the manufacturer's instructions. This array includes granulocyte-colony stimulating factor (G-CSF), monocyte chemotactic protein-1 (MCP-1), monokine induced by interferon-γ (MIG), macrophage inflammatory protein 1-α (MIP1-α) and macrophage inflammatory protein 1-β (MIP1-β).

Saliva samples were collected by expectoration in the morning by fasted participants. Samples were centrifuged at 1000 ***g*** for 10 min and supernatant fractions stored at –20°C before analysis. Salivary IgA content was determined by sandwich ELISA (Immunodiagnostik) in accordance with the manufacturer's instructions.

### Faecal sample processing

Freshly voided faecal samples were collected in sterile plastic containers. Samples were stored at room temperature under anaerobic conditions before processing, which typically commenced within 4 h of sample receipt. Samples for use in faecal dry weight and IgA assays were stored at –20°C, and samples for quantitative PCR and enumeration of total bacteria by flow cytometry stored at –80°C. Remaining faecal samples were diluted 1 in 10 (w/w) in PBS (0·1 m; pH 7·0) and homogenised in a Stomacher 400 (Seward) for 2 min at normal speed (460 paddle beats per min). A 15 ml sample of faecal slurry was vortexed with 2 g of 3-mm diameter glass beads (VWR International) and then centrifuged to remove particulate matter (1500 ***g***; 2 min). The supernatant fraction was collected for use in SCFA analysis and assessment of genus-level changes in the gut microbiota by fluorescence *in situ* hybridisation with 16S ribosomal RNA targeted oligonucleotide probes.

### Fluorescence *in situ* hybridisation

The faecal slurry supernatant fraction was fixed in paraformaldehyde (1:4 (v/v) in 4 % paraformaldehyde in 0·1 m-PBS, pH 7·2) for 4 h at 4°C, centrifuged (13 000 ***g***; 5 min), washed twice with 0·1 m-PBS, re-suspended in 1:1 PBS–ethanol and stored at –20°C. Oligonucleotide probes used were Cy-3 labelled and synthesised by Sigma-Aldrich. Probes used were Bif164, Bac303, Chis150, Lab158 and ATO291, Erec482, Fprau655, Enter1432 and Strc493 specific for *Bifidobacterium* spp., *Bacteroides–Prevotella* group, *Clostridium* clusters I and II (including *Clostridium perfringens* and *C. histolyticum*), *Lactobacillus–Enterococcus* subgroup, *Atopobium* cluster, *Eubacterium rectale*–*Blautia coccoides* group, *Faecalibacterium* cluster, *Enterobacterium* group and *Streptococcus* group–*Lactococcus*, respectively^(^^30^^–^^36^^)^. Samples were hybridised as described by Costabile *et al.*^(^^37^^)^. Data are expressed as log_10_ counts per g dry faeces.

### Quantification of *Bifidobacterium lactis*

DNA was extracted from the faecal samples with the use of the QIAamp DNA stool Mini kit (Qiagen) following the manufacturer's instructions. Quantitative PCR was used for quantification of *B. lactis* using the FAST SYBR green methodology (Applied Biosystems) in a total volume of 25 µl containing 1 ng of template DNA and 250 nm of the forward primer Blact_1^(^^38^^)^ and reverse primer Bflact5^(^^39^^)^. The amplification and detection of DNA were performed with an ABI 7500 sequencing detection system (Applied Biosystems). To obtain standard curves, a 10-fold dilution series ranging from 10 pg to 10 ng of DNA from the bacterial standard cultures was included in the PCR assays. For determination of DNA, triplicate samples were used, and the mean quantity per g wet weight was calculated.

### Organic acids

Filter-sterilised samples (1 ml) of faecal slurry (10 % (w/v) dilution of freshly voided faeces in PBS; pH 7·0) were used to determine faecal concentrations of organic acids including acetic acid, propionic acid, *i*-butyric acid, *n*-butyric acid, *i*-valeric acid, *n*-valeric acid, *n*-caproic acid and d-/l-lactic acid by HPLC. The column was an ion-exclusion REZEX-ROA organic acid column (7·8 × 300 nm; Phenomenex) maintained at 85°C. The eluent was 0·0025 mm-sulfuric acid in HPLC-grade water and the flow rate was 0·6 ml/min. Quantification of the samples was obtained through calibration curves of acetic acid, formic acid, propionic acid, butyric acid and lactic acid in concentrations between 6·25 and 100 mm.

## Results

### Subject characteristics

Recruitment of forty-one subjects for the study took place in the Reading area of the UK. Of these, forty volunteers passed the inclusion/exclusion criteria and started the trial in March 2008; thirty-seven completed the trial in October 2009. However, one subject was removed from the statistical analysis due to missing samples, resulting in a study size of thirty-six subjects (see the Sample size determination and randomisation section, Table 1 and Fig. 1).

### Analyses of baseline drift, carry-over effect and subject characteristics

Stability of the baseline was studied by comparing results from samples on day –5 (baseline) and on day 0 (before first supplementation). Using independent-samples *t* tests it was found that six out of forty parameters had significantly different levels (*P* < 0·05) on day 0 than on day –5 in some supplementation groups (Table 2). These results indicate that the baselines within the groups were not stable.

**Table 2.** Parameters having baseline drift or carry-over effects

Parameter	Treatment group	Baseline drift	Carry-over effect
*Bifidobacterium lactis*	Prebiotic		✓
*Bacteroides*–*Prevotella*	Probiotic		✓
*Blautia coccoides*–*Eubacterium rectale* cluster	Probiotic	✓	✓
Enterobacteria	Prebiotic	✓	✓
*Faecalibacterium prausnitzii* cluster	Synbiotic		✓
*Faecalibacterium prausnitzii* cluster	Probiotic	✓	
IL-8	Probiotic		✓
*Lactobacillus*–*Streptococcus* group	Probiotic		✓
Oxidative burst activity of monocytes	Probiotic		✓
Percentage of granulocytes having oxidative burst activity	Placebo	✓	
Percentage of monocytes having phagocytic activity	Placebo		✓
	Prebiotic	✓	✓
	Probiotic		✓
	Synbiotic	✓	
Phagocytic activity of monocytes	Placebo	✓	✓
	Probiotic		✓
Total bacteria	Probiotic		✓

Carry-over effects are a known problem in clinical trials with a cross-over design. Samples from day 0 (before first supplementation) and day 49 (after the first wash-out) were analysed for similarity using independent-samples *t* tests. By using *P* < 0·05 as a cut-off, it was found that in eleven out of forty parameters the values did not return to pre-supplementation levels in some groups (Table 2). It was concluded that carry-over effects might bias the results and thus statistical analysis was conducted for parallel groups using results from samples obtained on day 0 (before the first supplementation) and on day 21 (after the first supplementation).

The change in study design from cross-over to parallel resulted in an unbalanced age distribution between the study groups. Subjects in the maltodextrin and probiotic groups were of similar age, but subjects in the prebiotic and synbiotic groups were on average 6·8 years younger (*P* < 0·001) (Table 1).

### Activity of peripheral blood monocytes and granulocytes

**Fig. 2.**
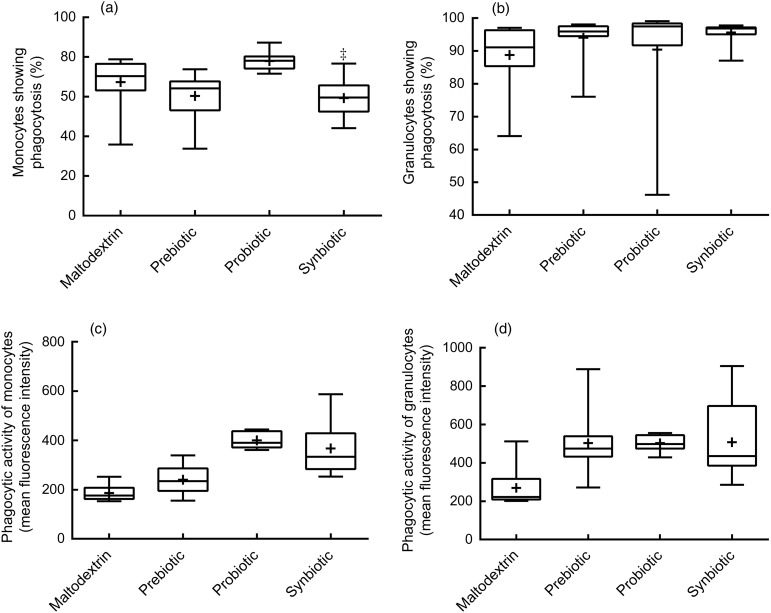
Phagocytic activity of monocytes and granulocytes. Phagocytic activity was measured using a flow cytometer after incubation of opsonised and fluorescence-labelled *Escherichia coli* in whole blood of the subjects. Percentage of monocytes (a) and granulocytes (b) of the total population that had phagocytosed *E. coli*. Phagocytic activity of monocytes (c) and granulocytes (d) measured as fluorescence intensity in individual cells that had phagocytosed *E. coli*. Statistical differences were calculated using linear model contrasts. Whiskers represent the minimum and maximum values; the box represents the 25th percentile, median and 75th percentile; + indicates the mean value. Mean value was significantly different from that of the maltodextrin group: *P = 0·02, **P < 0·001. Mean value was significantly different from that of the prebiotic group: †*P* = 0·005, ††*P* < 0·001.

The supplementations did not change the percentages of monocytes and granulocytes engaged in phagocytosis compared with maltodextrin (Fig. 2(a) and 2(b)); however, there was a higher percentage of phagocytosing monocytes in the probiotic group compared with the prebiotic (*P* = 0·005) group (Fig. 2(a)). Phagocytic activity of monocytes in the probiotic group was higher than in the maltodextrin (*P* < 0·001) or prebiotic groups (*P* < 0·001) (Fig. 2(c)). Furthermore, a higher phagocytic activity of granulocytes was found in the probiotic (*P* = 0·02) supplementation group compared with the maltodextrin group (Fig. 2(d)).

**Fig. 3.**
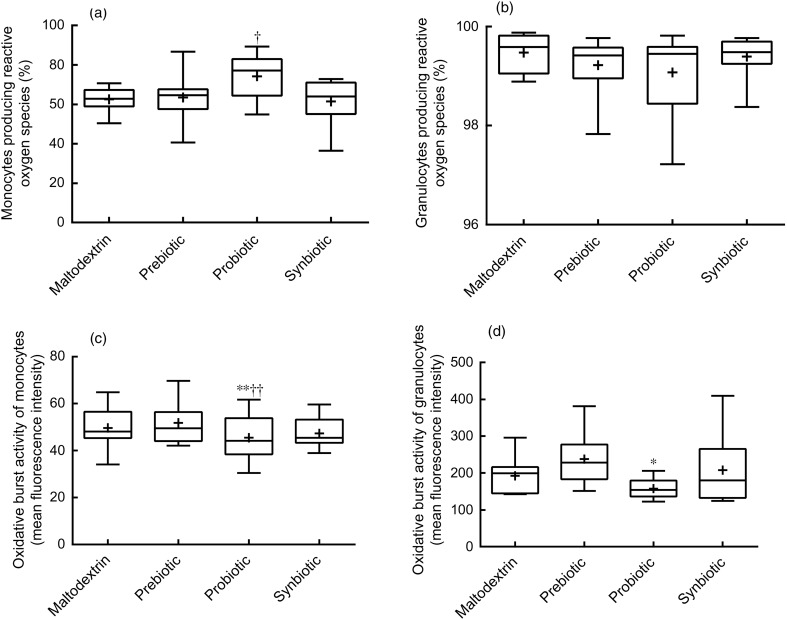
Oxidative burst activity of phagocytes. Oxidative burst activity was measured using a flow cytometer after incubation of opsonised and fluorescence-labelled *Escherichia coli* in whole blood of the subjects. Percentage of monocytes (a) and granulocytes (b) of the total population showing oxidative burst activity. Oxidative burst activity of monocytes (c) and granulocytes (d) measured as fluorescence intensity in individual cells that had oxidative burst activity. Statistical differences were calculated using linear model contrasts. Whiskers represent the minimum and maximum values; the box represents the 25th percentile, median and 75th percentile; + indicates the mean value. ‡Mean value was significantly different from that of the probiotic group (*P* = 0·04).

The BURSTTEST^®^ assay was used to measure the presence of ROS in phagocytes after incubation of opsonised *E. coli* in whole blood from the study subjects. The proportion of monocytes and granulocytes producing ROS was not affected by the supplementations compared with maltodextrin (Fig. 3(a) and 3(b)), nor was oxidative burst activity of these cells (Fig. 3(c) and 3(d)). However, the percentage of monocytes producing ROS in the probiotic group was higher than in the synbiotic group (*P* = 0·04) (Fig. 3(a)).

### Lipopolysaccharide stimulation of the whole blood and soluble immune markers

Immune markers were analysed from saliva, plasma, and by stimulating heparinised whole blood with LPS. The levels of cytokines in whole blood, chemokine concentrations in plasma and IgA levels in saliva were unaltered by the treatments (online Supplementary Table S1).

### Composition and metabolic function of the intestinal microbiota

Microbiota compositions of the subjects were characterised using 16S ribosomal RNA targeted fluorescence *in situ* hybridisation for the selected marker species or quantitative PCR for the determination of *B. lactis* (Table 3). Composition of the microbiota did not significantly change in any of the treatment groups. Metabolic activity of the microbiota was measured by analysing organic acid concentrations in the faeces, but there was no significant effect of any of the treatments (online Supplementary Table S2).

**Table 3.** Gut microbiota of volunteers during treatment with placebo, a prebiotic (galacto-oligosaccharides; GOS; 8 g/d), probiotic (*Bifidobacterium animalis* subsp. *lactis* Bi-07 (Bi-07); 10^9^ colony-forming units/d) or synbiotic (GOS + Bi-07) at the end of the first 21 d treatment period (Mean values with their standard errors)

	Treatment							
	Maltodextrin (*n* 9) (log_10_ cells/g faeces)		Prebiotic (*n* 9) (log_10_ cells/g faeces)		Probiotic (*n* 8) (log_10_ cells/g faeces)		Synbiotic (*n* 10) (log_10_ cells/g faeces)	
Bacterial group	Mean	sem	Mean	sem	Mean	sem	Mean	sem
*Atopobium* cluster	10·3	0·10	10·2	0·10	10·1	0·05	10·1	0·06
*Bacteroides*–*Prevotella*	10·6	0·07	10·4	0·15	10·6	0·08	10·4	0·08
*Bifidobacterium* group	10·1	0·13	9·7	0·20	9·4	0·39	9·9	0·13
*Bifidobacterium lactis*	5·7	0·25	6·0	0·22	6·3	0·22	6·2	0·30
*Clostridium* clusters I and II	8·8	0·24	8·1	0·27	8·9	0·43	7·9	0·27
*Blautia coccoides*–*Eubacterium rectale* cluster	10·7	0·07	10·6	0·10	10·8	0·06	10·5	0·06
Enterobacteria	9·5	0·31	9·1	0·27	9·2	0·41	9·4	0·17
*Faecalibacterium prausnitzii* cluster	10·5	0·05	10·3	0·12	10·5	0·04	10·4	0·09
*Lactobacillus*–*Streptococcus* group	9·2	0·24	8·6	0·14	9·4	0·16	8·6	0·10
Total bacteria	11·3	0·09	11·2	0·12	11·3	0·07	11·2	0·06

## Discussion

A randomised placebo-controlled cross-over clinical trial was designed to study the effect of probiotic Bi-07, prebiotic GOS or their synbiotic combination Bi-07 + GOS on gut and immune functions in healthy non-institutionalised elderly adults. It was found that the baseline of some of the parameters drifted before supplementation (Table 2), which could indicate natural fluctuation in this population. In addition, statistical analyses of the cross-over study revealed significant carry-over effects on indices of microbiota and immune function (Table 2), indicating that a 4-week wash-out period was not sufficient in this trial. In a similar study set-up, the effect of GOS syrup (8·1 g GOS/d), *B. lactis* Bb-12 and their combination was studied on bifidobacteria numbers in healthy adults. It was found that Bb-12 and Bb-12 + GOS syrup supplementation increased bifidobacteria amounts, but the levels returned to normal after a 2-week wash-out period^(^^24^^)^. Another study has also shown that after 2 weeks of wash-out, Bb-12 could be recovered from the faeces of only a few individuals, indicating transient colonisation of *B. lactis* in healthy adults^(^^40^^)^. Furthermore, two recent studies utilising pyrosequencing showed that 2 weeks after GOS supplementation the microbiota composition returned to baseline levels in healthy adults^(^^41^^,^^42^^)^. Taken together, the results of these studies suggest that a 2-week wash-out period is long enough in healthy adults. In the present study, however, an elderly population was studied and it may be that probiotic and prebiotic supplementation induces longer-lasting changes in elderly microbiota than in adults. Interestingly, in a series of papers, elderly subjects were supplemented with *B. lactis* HN019 or *Lactobacillus rhamnosus* HN001 and after a 3-week wash-out period several immune indices were numerically higher than at baseline; however, unfortunately, wash-out time points were not statistically compared with baseline^(^^25^^,^^43^^,^^44^^)^.

To eliminate cross-over effects, samples from only the first supplementation period were used in the subsequent statistical analyses, making the statistical analysis design parallel instead of cross-over. This resulted in a higher average age in the maltodextrin and probiotic groups compared with in the prebiotic and synbiotic groups. As it is known that microbiota composition and immunological functions decline during ageing, the prebiotic and synbiotic efficacy (or not) is merely speculative in the present study. However, the comparison of the maltodextrin control with the probiotic seems to be valid.

It was found that consumption of Bi-07 improved the phagocytic activity of monocytes and granulocytes against complement and immunoglobulin-opsonised *E. coli* (Fig. 2(c) and 2(d)). In previously published clinical trials, enhanced phagocytosis in healthy elderly adults has been shown for *Bifidobacterium animalis* subsp. *lactis* HN019^(^^25^^,^^45^^)^ and also in healthy adults for strains Bb-12 and HN019 of the same species^(^^46^^,^^47^^)^. These studies support the results observed for Bi-07 in the present trial and indicate a positive effect of *B. lactis* consumption on phagocytic activity in general that could potentially lead to improved immune responses against infective agents.

Although improved phagocytic activity was observed in the probiotic group (Fig. 2(c) and 2(d)), it did not lead to higher intracellular ROS generation than what was observed for the maltodextrin group (Fig. 3(c) and 3(d)). This result was supported by similar pro-inflammatory cytokine response levels to LPS (TNF-α, IFN-γ, IL-1β, IL-6, and IL-8) and stable chemokine levels in plasma (online Supplementary Table S1) in the maltodextrin and probiotic groups. On the other hand, probiotic supplementation did not improve anti-inflammatory IL-10 production either (online Supplementary Table S1). Previous *in vitro* studies have shown that in comparison with other *Bifidobacterium* and *Lactobacillus* strains, Bi-07 is among the most potent inducers of IL-12p70 and IFN-γ from human peripheral blood monocytes^(^^48^^)^. IFN-γ and IL-12p70 are prototypical cytokines involved in activating Th1-type immune responses that enhance phagocytosis and intracellular killing of microbes. On the other hand, Bi-07 also induced a moderate amount of IL-10 from peripheral blood mononuclear cells and protected mice from trinitrobenzene sulfonic acid (TNBS)-induced colitis^(^^48^^)^, which could explain the null impact on ROS generation. This type of non-inflammatory phagocytosis is an important mechanism of maintaining homeostasis in the gut mucosa where overt reactions to commensal microbes may lead to conditions such as inflammatory bowel disease.

In the present randomised clinical trial on healthy elderly adults, the probiotic Bi-07 had a positive impact on the phagocytic activity of monocytes and granulocytes in the elderly subjects without increasing the release of ROS. Consumption of Bi-07 could potentially improve clearance of bacteria from the body without contributing to the low-grade inflammation observed in the elderly population^(^^49^^)^. Thus, consumption of the probiotic Bi-07 may provide long-term health benefits for the elderly by not contributing to inflammation-associated metabolic disorders while enhancing the innate immune defence against infections.

The present results indicate that the wash-out time of supplementation may need to be longer in elderly subjects than what has been shown for adults. Overall, it is likely that the effective time of the probiotics and prebiotics on the gut microbiota and immune function is dependent on the population, diet, and on the properties of the probiotics and prebiotics themselves. It is clear that well-designed clinical trials on the effects of probiotics, prebiotics and synbiotics on health are acutely needed.

## Supplementary Material

Supplementary Material Supplementary information supplied by authors.

## Supplementary material

To view supplementary material for this article, please visit http://dx.doi.org/10.1017/jns.2013.31
