# Consumption of *Bifidobacterium lactis* Bi-07 by healthy elderly adults enhances phagocytic activity of monocytes and granulocytes – ERRATUM

**DOI:** 10.1017/jns.2014.1

**Published:** 2014-02-28

The publishers regret to announce that the symbols in Figs. 2 and 3 were incorrectly published in Maneerat *et al*.^(^^1^^)^.

**Fig. 2.**
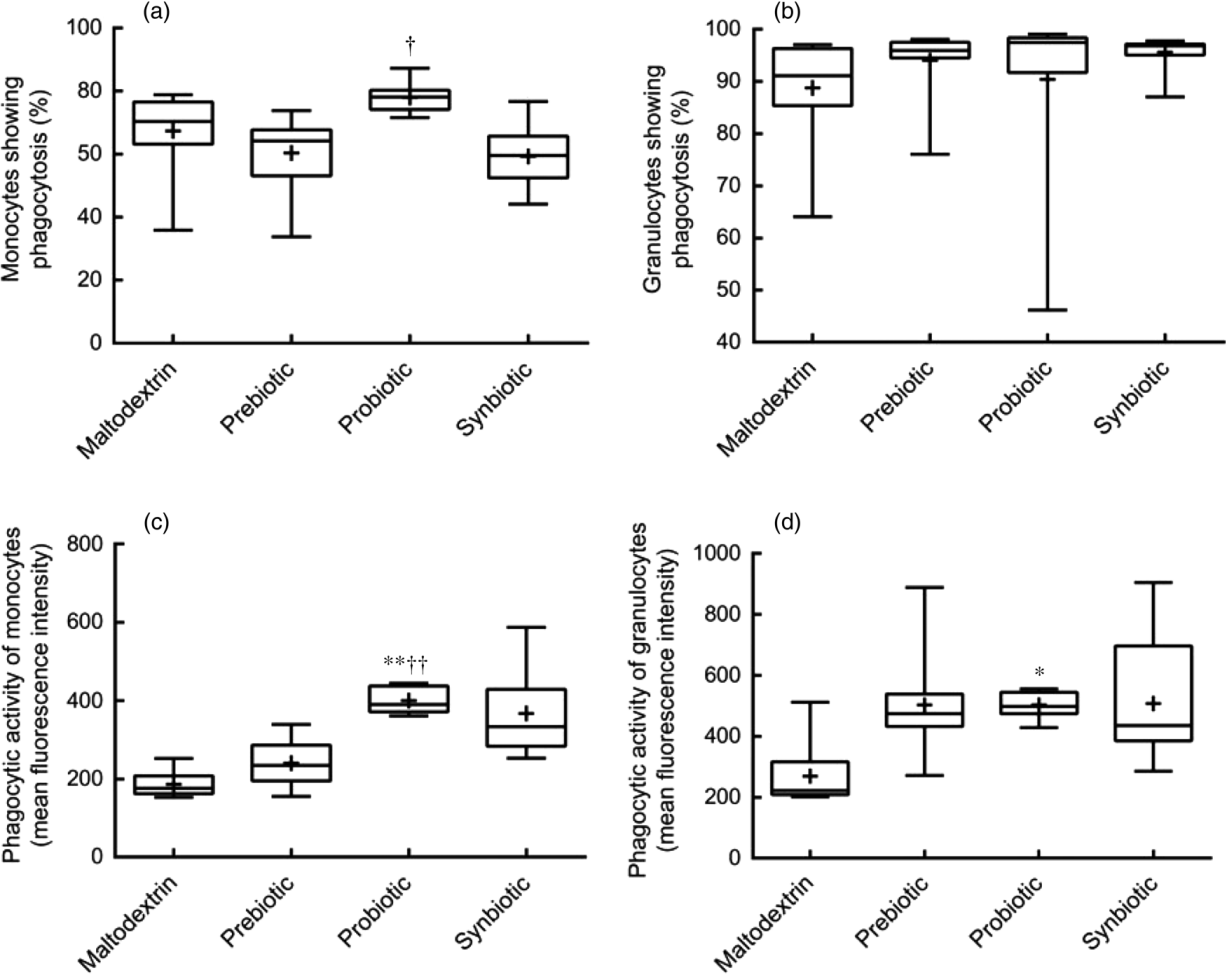
Phagocytic activity of monocytes and granulocytes. Phagocytic activity was measured using a flow cytometer after incubation of opsonised and fluorescence-labelled *Escherichia coli* in whole blood of the subjects. Percentage of monocytes (a) and granulocytes (b) of the total population that had phagocytosed *E. coli*. Phagocytic activity of monocytes (c) and granulocytes (d) measured as fluorescence intensity in individual cells that had phagocytosed *E. coli.* Statistical differences were calculated using linear model contrasts. Whiskers represent the minimum and maximum values; the box represents the 25th percentile, median and 75th percentile; + indicates the mean value. Mean value was significantly different from that of the maltodextrin group: *P = 0·02, **P < 0·001. Mean value was significantly different from that of the prebiotic group: †*P* = 0·005, ††*P* < 0·001.

**Fig. 3.**
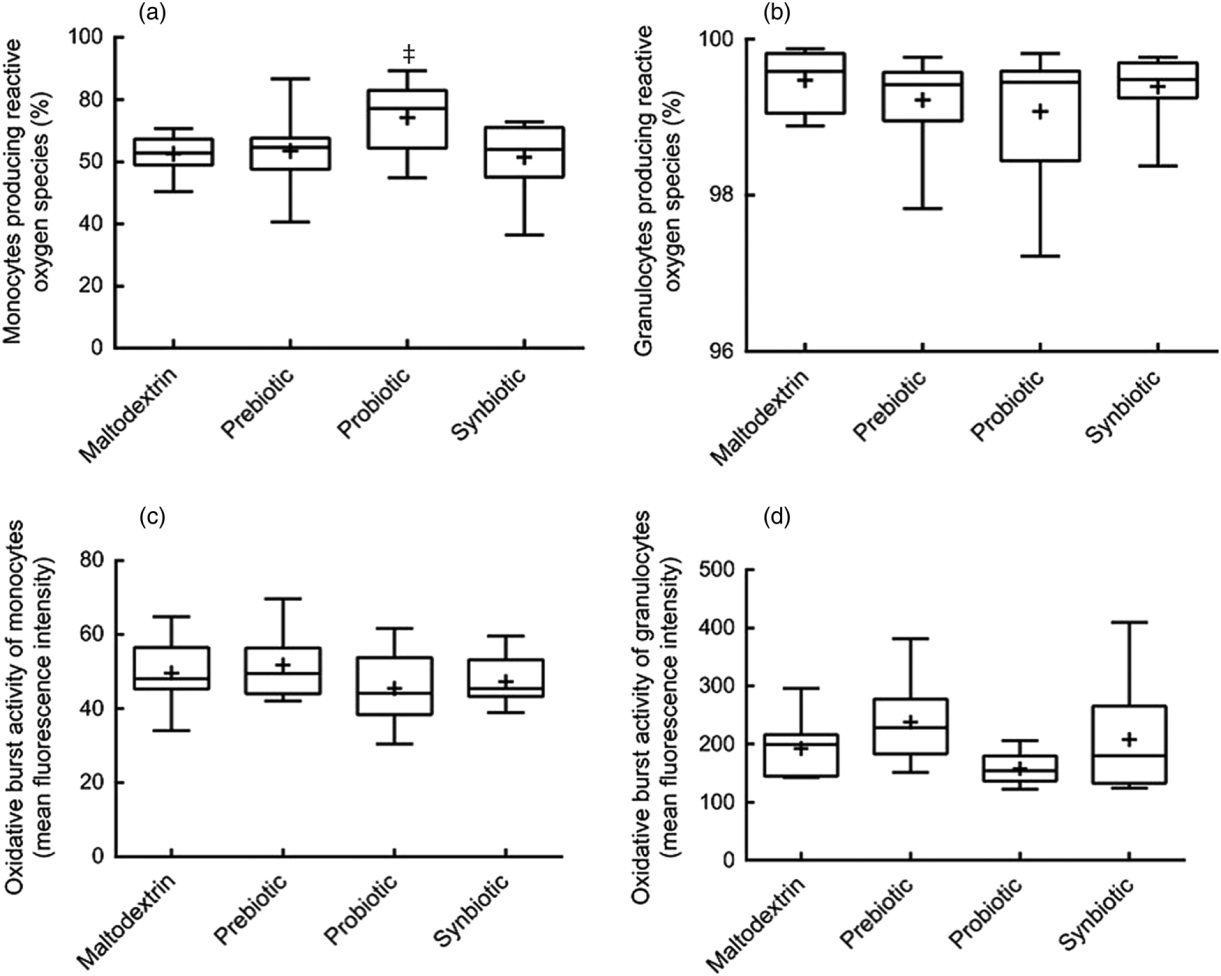
Oxidative burst activity of phagocytes. Oxidative burst activity was measured using a flow cytometer after incubation of opsonised and fluorescence-labelled *Escherichia coli* in whole blood of the subjects. Percentage of monocytes (a) and granulocytes (b) of the total population showing oxidative burst activity. Oxidative burst activity of monocytes (c) and granulocytes (d) measured as fluorescence intensity in individual cells that had oxidative burst activity. Statistical differences were calculated using linear model contrasts. Whiskers represent the minimum and maximum values; the box represents the 25th percentile, median and 75th percentile; + indicates the mean value. ‡Mean value was significantly different from that of the synbiotic group (*P* = 0·04).

The figures as they should have appeared, along with their correct captions, are printed below. The publishers apologise for this error.
