# Supplementary material for: Consumption of Bifidobacterium lactis Bi-07 by healthy elderly adults enhances phagocytic activity of monocytes and granulocytes
Source: J Nutr Sci. 2014 Jan 2;2:e44. doi: 10.1017/jns.2013.31 (PMC4153077; doi:10.1017/jns.2013.31)
Supplement: Supplementary Material — Supplementary information supplied by authors. [file S2048679013000311sup001.docx]

**Online supplementary material**

**Supplementary Table S1.** Cytokine production of whole blood cultures stimulated with LPS *in vitro*, plasma chemokines and salivary IgA among volunteers recruited to a double-blind, placebo-controlled, randomised cross-over during treatment with a prebiotic (GOS, 8g/day), probiotic (*Bifidobacterium animalis* subsp *lactis*, Bi-07, 10^9^ CFU/day) or synbiotic (GOS + Bi-07) ^1^. Statistical differences were calculated using ANOVA and 2x2 factorial design.

|  | |  |  | Treatment |  |  |  |  |  |
| --- | --- | --- | --- | --- | --- | --- | --- | --- | --- |
|  | | **Maltodextrin**  **(n=9)** | | **Prebiotic**  **(n=9)** | | **Probiotic**  **(n=8)** | | **Synbiotic**  **(n=10)** | |
| *Cytokines (pg/ml)* | | **Mean** | **SEM** | **Mean** | **SEM** | **Mean** | **SEM** | **Mean** | **SEM** |
| IFN-γ | | 768 | 166 | 946 | 332 | 1861 | 466 | 1826 | 700 |
| IL-1β | | 1445 | 271 | 1351 | 225 | 1161 | 210 | 1836 | 484 |
| IL-6 | | 4024 | 874 | 4874 | 534 | 4271 | 736 | 4447 | 763 |
| IL-8 | 5448 | 931 | 4782 | 688 | 2857 | 491 | 4249 | 760 |  |
| IL-10 | | 462 | 86 | 602 | 84 | 364 | 83 | 393 | 58 |
| TNF-α | | 1602 | 834 | 1421 | 451 | 807 | 171 | 1068 | 391 |
| *Plasma chemokines (pg/ml)* | |  |  |  |  |  |  |  |  |
| G-CSF | | 356 | 207 | 295 | 152 | 323 | 110 | 728 | 243 |
| MCP-1 | | 547 | 39 | 589 | 47 | 672 | 88 | 588 | 41 |
| MIG | | 360 | 65 | 352 | 93 | 278 | 17 | 557 | 150 |
| MIP-1α | | 599 | 270 | 614 | 328 | 1049 | 401 | 2469 | 1561 |
| MIP-1β | | 55 | 20 | 49 | 14 | 46 | 16 | 56 | 29 |
| *Salivary antibodies (µg/ml)* | |  |  |  |  |  |  |  |  |
| IgA | | 1914 | 519 | 1164 | 242 | 1594 | 459 | 1199 | 197 |

**Supplementary Table S2. Concentrations of short-chain fatty acids in the faeces.** Concentrations of short-chain fatty acids were analyzed from the feces of the subjects using High Performance Liquid Chromatography.^1^ Other organic acids such as lactic and formic acids were not detected. Statistical differences were calculated using ANOVA and 2x2 factorial design.

|  |  |  |  | Treatment | |  |  |  |
| --- | --- | --- | --- | --- | --- | --- | --- | --- |
|  | **Maltodextrin (n=9)**  **(mmol/l)** | | **Prebiotic (n=9)**  **(mmol/l)** | | **Probiotic (n=8)**  **(mmol/l)** | | **Synbiotic (n=10)**  **(mmol/l)** | |
| *Short-chain fatty acid* | **Mean** | **SEM** | **Mean** | **SEM** | **Mean** | **SEM** | **Mean** | **SEM** |
| Acetic acid | 5.5 | 0.7 | 5.3 | 0.9 | 5.2 | 0.7 | 5.5 | 0.5 |
| Propionic acid | 1.7 | 0.3 | 1.5 | 0.3 | 1.4 | 0.2 | 1.4 | 0.2 |
| Butyric acid | 6.0 | 1.6 | 4.9 | 1.3 | 6.5 | 1.4 | 4.1 | 1.0 |
